# Scots Pine Bark Extracts as Co-Hardeners of Epoxy Resins

**DOI:** 10.3390/molecules30010065

**Published:** 2024-12-27

**Authors:** Tomasz Szmechtyk

**Affiliations:** Department of Physical Chemistry, Faculty of Chemistry, University of Łódź, Pomorska 163/165, 90-236 Łódź, Poland; tomasz.szmechtyk@chemia.uni.lodz.pl

**Keywords:** epoxy, crosslinking agent, DSC, ATR FT-IR, UV-VIS, pine bark extract

## Abstract

Extracts from natural waste like bark or leaves are great sources of phytochemicals, which contain functional groups (hydroxyl, carboxylic, vinyl, allyl) attractive in terms of polymer synthesis. In this study, the synthesis of epoxy with an extract of Scots pine bark as a natural co-hardener was evaluated. Ultraviolet-visible (UV-Vis) spectroscopy was used for the identification of phytochemicals with conjugated dienes and quantification of TPC. Also, the total solid content (TSC) of representative extracts was calculated. The best extract in terms of total phenolic content (TPC) value was selected as a co-hardener and investigated using differential scanning calorimetry (DSC) for thermal effects and attenuated total reflectance Fourier transform infrared spectroscopy (ATR FTIR) for reactions between functional groups. Also, the mechanical properties (flexural modulus, flexural strength, impact strength, Shore D hardness) and density of composition were obtained for extract-based epoxy and compared to reference sample values. Results were discussed in terms of future research and improvement of compositions. Also, potential applications were proposed.

## 1. Introduction

Epoxy resins are common thermosets with a wide range of applications. Despite this, the majority of commercial epoxies are not green and completely safe materials. Substrates like bisphenol A (BPA) and curing agents are toxic. Even hardened resin can release harmful BPA [1,2,3]. All aforementioned substrates are also petroleum-based.

Current research focuses on overcoming these limitations by adding natural substances to epoxy compositions. The oldest and most well-known solution is the addition of fillers of natural origin. These fillers are usually finely ground and act as active or semi-active additives that improve the properties of thermosetting resins [4]. Powdered barks of tropical trees and shrubs like mesquite (*Prosopis juliflora*) [5], *Sterculia foetida* [6], and neem (*Azadirachta indica*) [7] were recently tested as fillers. Also, the barks of birch (*Betula* sp.) [8] and spruce (*Picea* sp.) [9,10] were parts of newly reported epoxy compositions.

Epoxy laminates have been reinforced with natural bast fibres for years. The most well-known and, despite this, still modified and tested are flax [11], jute [12], kenaf, hemp, and ramie. Sahu et al. tested *Calotropis gigantea* fibres after alkali treatment as a lightweight alternative for commercial reinforcements in the automotive industry [13]. Eltahir et al. proposed baobab (*Adansonia digitata* L.) fibres as natural reinforcement with improved thermal stability (250 °C) [14]. Balasubramanian et al. reported epoxy with bark fibres from Indian elm (*Holoptelea integrifolia*) [15]. Recently, natural fibres are also mixed with inorganic ones for unique properties. Jeyaguru et al. proposed a hybrid woven of hemp with Kevlar as reinforcement to obtain an inexpensive alternative to high-performance synthetic fibres in terms of water absorption, thickness swelling, and erosion resistance [16]. Another added value is the design of different weaving architectures. Twill-waved abacá (*Musa textilis*) fibres and bi-directional basalt fibres were stacked alternately in an epoxy laminate for improved compressive and damping properties [17].

Another “green option” is epoxidation of phytochemicals with epichlorohydrin. Unlike the quite popular linseed oil and soybean oil, extracts from barks are not commonly used as substrates. Zhang et al. prepared epoxy from myrica (*Myrica* sp.) tannin extract through liquefaction and epoxidation, which was added to bisphenol A diglycidyl ether (BADGE) resin as modifier (enhanced mechanical properties) [18]. Shnawa epoxidised extract from eucalyptus (*Eucalyptus* sp.) bark and blended it with commercial epoxy, but only blends with 20% tannin epoxy were competitive [19]. Another solution was proposed by Zhu et al.—epoxy obtained from bog-myrtle (*Myrica gale*) bark extract was copolymerised with chitosan and applied as a protective coating for paper [20]. Promising results were also obtained in studies with bark extracts of houpu magnolia (*Magnolia officinalis*) and commercially separated magnolol. Self-curing behaviour of such epoxy [21], improved stiffness for composition cross-linked with amine hardener [22], reinforcement of chitosan-based aerogel [23], and reactive flame-retardant (diglycidyl ether of magnolol phosphine oxide) [24] were reported. Chemical modification of phytochemicals is also a method of obtaining curing agents. Xu et al. [25] synthesised a tannic acid derivative with carboxylic groups as a hardener for epoxidised gallic acid.

The purified tannic acid (TA), also an unmodified substance, was used as a curing agent for epoxidised aliphatic compounds [26,27,28] and commercial BADGE resin [29]. Best results were obtained for compositions of epoxidised linseed oil (ELO) with high content of TA (molar ratio of ELO to TA—1.75 and 2.0)—flexural modulus over 2 GPa and flexural strength over 65 MPa [29]. Also, the epoxidised soybean oil (ESO) with TA had better properties for increased hydroxyl to epoxy ratio (1.4)—tensile modulus: 458 MPa, tensile strength: 15.1 MPa [27]. Bo et al. used TA as a co-hardener with m-phenylenediamine to obtain crosslinked resin based on tetraglycidyl-4,4′-methylene dianiline [30]. For composition with mass ratio 3 between TA and epoxy, the best tensile strength (112.2 MPa) and toughness (2.21 MPa) were obtained. The commercial mixture of gallic tannins from tara (*Caesalpina spinosa*) with over 90% of tannic acid content was used as an additive, improving the adhesion of UV-curable epoxy (epoxidised soybean oil with photoinitiator) but the presence of TA decreased thermo-mechanical properties and hardness of coatings [31]. Montoya et al. [32] extracted polyphenols from Monterey pine (*Pinus radiata*) and added small amounts of them to epoxy-amide coatings as corrosion inhibitors. Two compositions with extract fractions, water-soluble (WSF) and water-insoluble (WIF), were relatively competitive with commercial coating (CC), showing slightly lower adherence (WSF: 8.1 MPa and WIF 7.0 MPa compared to 10.4 MPa of CC), lower dynamic viscosity (WSF: 2552 cP and WIF 4368 cP compared to 5096 cP of CC), and slightly lower Shore A hardness (WSF: 38.4 and WIF 38.5 compared to 42.4 of CC).

Herein, the application of unmodified Scots pine (*Pinus sylvestris*) bark extract as a co-hardener for epoxy resin with Mannich base hardener is reported. Pine bark extract, as substrate obtained from nature, was tuned in terms of optimal extraction conditions (total phenolic content and ultraviolet-visible (UV-Vis) spectroscopy identification of phytochemicals with conjugated dienes). Also, the total solid content (TSC) of representative extracts was calculated. According to the total phenolic content (TPC) value, one extract was mixed as a co-hardener with commercial epoxy and hardener. The curing process of composition was investigated using differential scanning calorimetry (DSC) and attenuated total reflectance Fourier transform infrared spectroscopy (ATR FTIR). Mechanical properties were obtained from Shore D hardness, three-point flexural test, and Charpy impact test. Additionally, densities of compositions for checking porosity were measured. The obtained results were extensively discussed.

## 2. Results

### 2.1. Total Phenolic Content Analysis

Scots pine bark was collected, grounded, and extracted. The total phenolic content (TPC) of extracts was measured and expressed as gallic acid equivalent (GAE). The TPC results are presented in Table 1. The highest amount of polyphenols (3670.2 ± 68.4 µg GAE/mL) was obtained for extract C120 (conventional extraction, 120 min), while extract U15 (ultrasound-assisted extraction, 15 min) had the lowest TPC value (1981.1 ± 58.5 µg GAE/mL). Surprisingly, the C90 extract shows a relatively low TPC value compared to adjacent C60 and C120. It could be the result of light-induced degradation and oxidation (samples were prepared in the air) of catechins [33,34]. Also, the anthocyanins are sensitive to high temperatures, and this extraction time could be crucial for their stability [35]. Probably, these lost phenolic compounds were quite large molecules because in pilot tests (separation of bark extract by filtration instead of centrifugation, worse TPC results), they were left on the filter, and no significant decrease of C90 TPC value was observed. On the other hand, 2 h of extraction could cause an increase of phenolic acids that were bound to lignin [36] and result in a high TPC value for the C120 extract. Similar mechanisms (degradation and cross-linking induced by ultrasounds) could be the reason for the nonlinear TPC—time relation for extracts U30, U45, and U60. Also, the temperature increase as a side effect of the UAE process could be crucial. Both mixed extractions (U+C) did not provide outstanding results. Their TPC values are higher than values of single components alone but still not competitive (the TPC of more economically justified C30 extract is higher). These results also confirm that C90 decrease (similar to low TPC improvement from U30 to U30+C90) and C120 decrease (similar to 500 µg GAE/mL growth between TPCs of U15 and U15+C105) are not single cases. C120 extract was selected for further tests.

### 2.2. Total Solid Content

Four representative extracts (C30, C120, U30, and U30+C90) were selected for the determination of total solid content (TSC). The results are presented in Table 2. The highest TSC value was obtained for the U30+C90 extract, while the C30 extract had the lowest amount of solids after methanol evaporation. U30 and C120 had similar TPC values.

### 2.3. UV-Vis Identification of Phytochemicals

**Figure 1:**
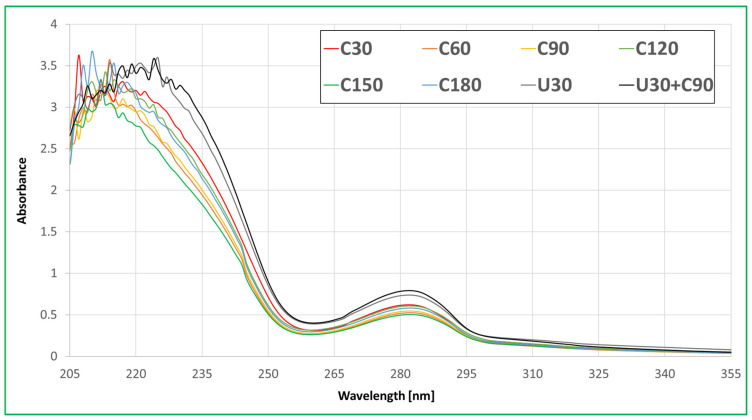
UV-Vis spectra of selected eight representative extracts.

Compared UV-Vis spectra of selected eight representative extracts (Figure 1) show similar characteristics, suggesting that the chemical compositions of these extracts are based on related compounds. The main absorption bands were observed in the broad range of 205 to 250 nm. The presence of multiple peaks indicates at least a few factors contributing to this band. Peaks around 208 and 210 nm are related to max. absorption of β-pinene and α-pinene, respectively [37]. Both of these are the most abundant volatile compounds found in Scots pine bark [38]. Other contributing factors for this band are absorption maxima of polyphenolic C-rings without conjugated diene inside (hypsochromic shift) [39]. The groups of such polyphenols are dihydroflavonols (taxifolin) [32], flavanones, flavan-3-ols (catechin) [32,38,40], and glycosides of all three, which are also confirmed by band of 260 to 300 nm [39]. Also, according to Karonen et al.’s study [41], other types of phenolic compounds reported in Scots pine bark (vanillin, β-hydroxypropiovanillone, ferulic acid, dihydroconiferyl alcohol, and pinoresinol) may also contribute to these both bands (their absorption maxima are in the ranges). Both spectra of UAE representatives (U30 and U30+C90) have increased absorption of band 260 to 300 nm and peak shift from 210 nm to 225 nm for the 205–250 nm band. This indicates a higher content of phenolic compounds compared to conventional extracts.

### 2.4. DSC Analysis of Curing Process

**Figure 2:**
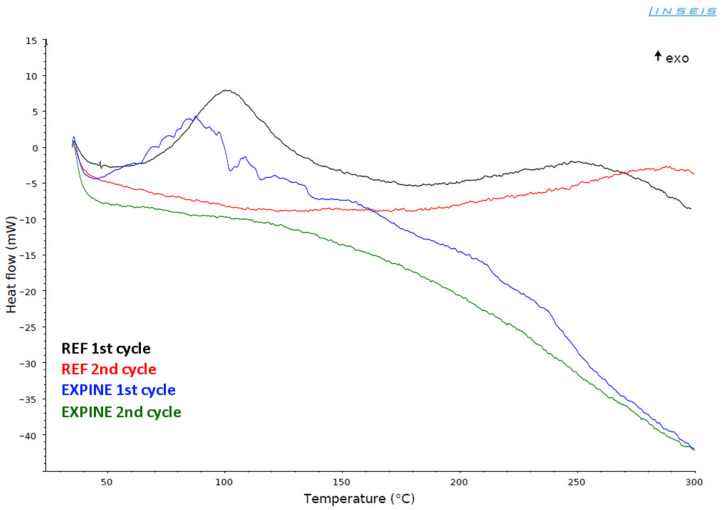
DSC curves of REF and EXPINE samples—two cycles of curing from 35 °C to 300 °C.

Differential scanning calorimetry (DSC) was used as a comparison tool for curing characteristics of the reference sample (REF) and composition with selected C120 pine extract (EXPINE). DSC curves of REF and EXPINE samples were compared in Figure 2. The first curing cycle of REF composition has two exothermic effects, typical for curing processes of epoxy compositions with complex chemistry, like epoxy with Mannich base [42,43]. The first effect starts from 55 °C (peak around 100 °C), ends around 180 °C and mainly results from reactions of epoxy groups with amine groups (primary over secondary amines) and then phenolic groups (which are catalysed by forming tertiary amines) from TFF hardener [44]. The second effect starts immediately from 180 °C (peak at 250 °C, more flat than the peak at 100 °C) and ends around 300 °C. It is the effect of more sluggish post-curing reactions between remaining secondary amines and remaining epoxy groups or secondary hydroxyl groups from previous reactions of epoxy groups [45,46]. The second curing cycle of REF provides only one exothermic effect (post-curing) starting from around 200 °C with a peak at 290 °C.

**Figure 3:**
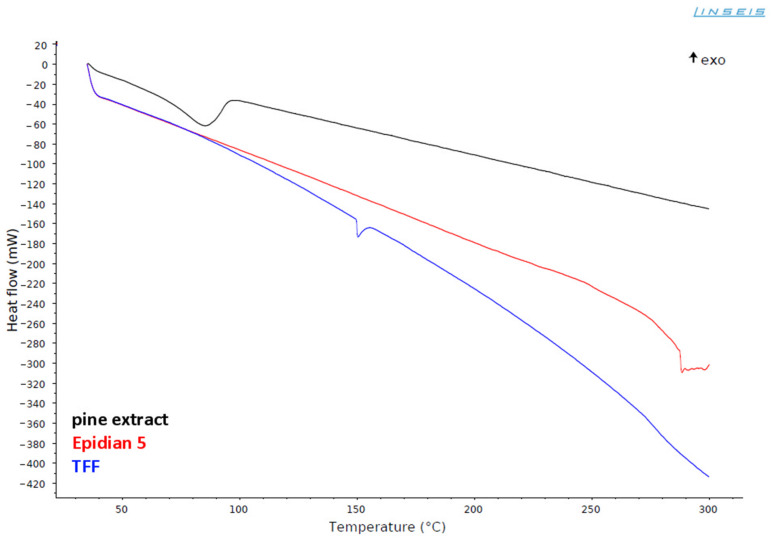
DSC curves of substrates—cycle from 35 °C to 300 °C.

EXPINE composition has even more complex chemistry as a result of the presence of various phytochemicals from pine extract. The first curing cycle is a reflection of this complexity. The first exothermic effect (curing reaction) starts around 40 °C, but it has a more rough line than the corresponding peak of the REF curve. It is a result of methanol evaporation [47], which has an intermittent nature due to the gelation of composition. Convergence of these processes could result in increased porosity of final material compared to REF [47]. The highest peak is observed around 90 °C, which indicates that the curing reaction is catalysed to some extent (compared to the corresponding process of REF). According to Shechter et al. [44], phenolic compounds from pine extract act as catalysts for epoxy-amine reactions. As in the case of REF composition, hydroxyl groups of aromatic moieties (from phytochemicals and TFF) start reactions with epoxy groups after forming tertiary amines. The first exothermic effect ends around 140 °C. According to the DSC curve of pure pine extract (Figure 3), methanol should evaporate completely slightly after 100 °C, even in partially cured resin with limited permeability. It corresponds to reduced roughness of EXPINE 1st cycle curve after 120 °C. The second exothermic effect starts immediately at 140 °C in the same way as for REF (secondary functional groups react with remaining epoxy rings) but differs from it by three, even more, flat peaks (at 155 °C, 210 °C and 235 °C). This difference indicates that post-curing is less chemically uniform, and more complex aromatic donors of phenolic groups react more sluggishly than phenol from TFF as a result of steric hindrance. On the other hand, the second effect ends around 260 °C, which indicates a lower availability of reactive groups, but also the presence of the extract’s catalytic effect. For both EXPINE cycles baseline decrease from 140 °C is observed. It corresponds to the end of the first curing peak and probably results from permanent lid opening by cured epoxy elevated by methanol.

### 2.5. ATR FTIR Analysis of Curing Process

**Figure 4:**
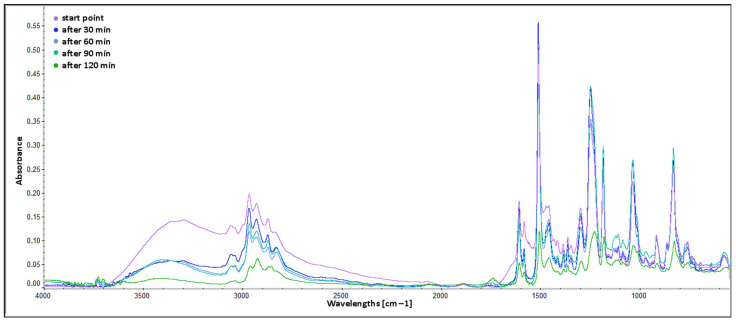
ATR FTIR spectra of BADGE epoxy cured with TFF and C120 extract—full range.

The spectra of EXPINE composition obtained at thirty-minute intervals were compared in the full range from 4000 to 550 cm^−1^ (Figure 4) and in smaller ranges, crucial for the analysis of the curing process. Analysis of spectra was mainly based on the Infrared Spectroscopy section from the Virtual textbook of organic chemistry by William Reusch [48]. The evaporation of methanol, observed from the start point spectrum, influences the absorbance of most of the bands, which is getting lower and peaks becoming sharper. Also, the peak around 1740 cm^−1^ (attributed to C=O stretching vibrations in esters) increased for “after 120 min” spectra. It results from the reaction between hydroxyl groups and carboxylic groups from polyphenolic acids (peak around 1700 for other spectra, overlapped).

**Figure 5:**
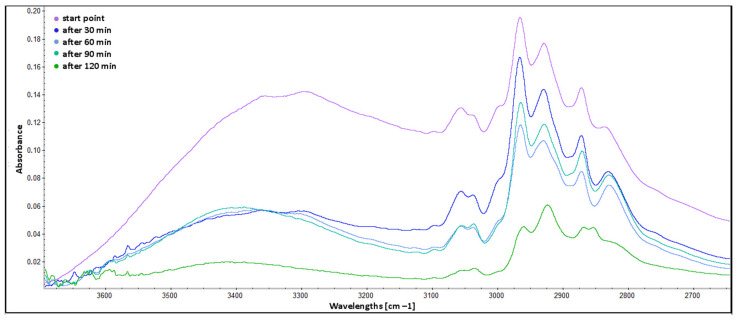
ATR FTIR spectra of BADGE epoxy cured with TFF and C120 extract—range from 4000 to 2650 cm^−1^.

The comparison of spectra in the range from 4000 to 2650 cm^−1^ is presented in Figure 5. The broadband from 3600 to 3200 cm^−1^ (stretching vibrations of hydroxyl groups) shows a significant decrease of absorbance between the “start point” and “after 30 min” spectra. This decrease is a result of the evaporation of most of the methanol, which occurred between these measurements. The peak of this broadband is shifted to 3350 cm^−1^ for “after 60 min” and to 3400 cm^−1^ for “after 90 min” spectra compared to “after 30 min” spectra (peak at 3300 cm^−1^). The shift is an effect of increased content of hydroxyl groups from phenolic compounds and epoxy groups, which have undergone a chemical reaction of ring opening. The bands from 3025 to 3100 cm^−1^ (assigned to =C–H stretching vibrations of alkene groups, mainly from pinenes) [38] also show decreased intensity and sharpness at the time of the curing process, which suggests reactions of such groups.

**Figure 6:**
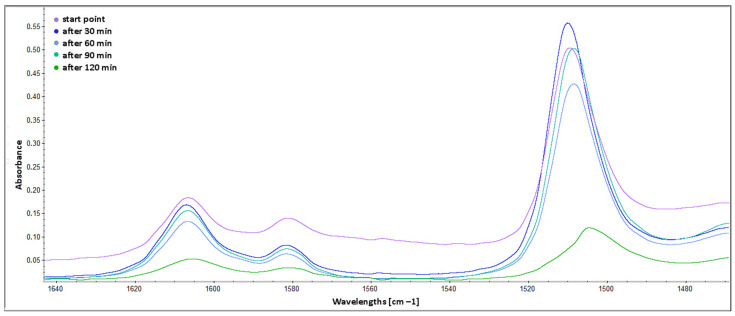
ATR FTIR spectra of BADGE epoxy cured with TFF and C120 extract—range from 1640 to 1470 cm^−1^.

The comparison of spectra in the range from 1640 to 1470 cm^−1^ is presented in Figure 6. The two bands from 1620 to 1590 cm^−1^ (in-plane NH_2_ scissoring vibrations of primary amine groups from TETA) have decreasing absorbance as a result of the curing process. It is proof that amine groups still work as epoxy crosslinkers. The peak around 1510 cm^−1^ (aromatic skeletal vibration of benzene ring) is a marker of the presence of various aromatic compounds, including lignin structures [49,50,51], which can be more easily extracted as a result of decomposition processes (the bark was taken from a fallen tree) [52]. The shift of the peak to 1505 cm^−1^ (after 90 min and 120 min spectra) and decrease of its absorbance (for “after 120 min” spectrum) could be a result of a more dense and rigid structure similar to guaiacyl–syringyl units of hardwoods [53,54]. Such structures are evidence that tannins and other compounds contained in the extract react with epoxy groups and participate in the formation of cross-linked resin.

**Figure 7:**
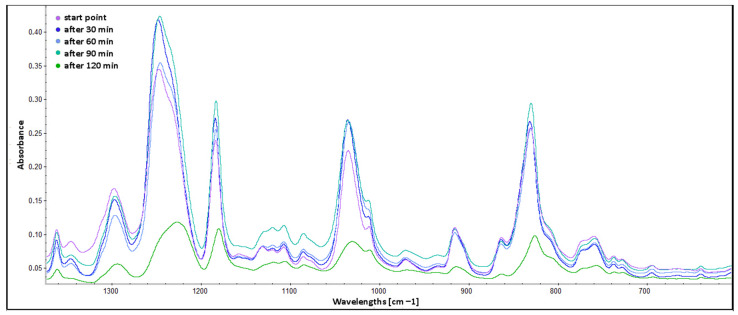
ATR FTIR spectra of BADGE epoxy cured with TFF and C120 extract—range from 1370 to 620 cm^−1^.

The comparison of spectra in the range from 1370 to 620 cm^−1^ is presented in Figure 7. The methylene twisting band with a peak at 1300 cm^−1^ shifts to 1295 cm^−1^ and has decreased intensity for “after 120 min” spectrum. This change is a result of the liquid-to-solid phase transition [55], which is a consequence of the curing process. The strong band with a peak at 1245 cm^−1^ is from the C–O bond in acetyl groups present in hemicelluloses [56] (xylans) and lignins [57], especially those degraded by fungi [58,59]. The 1245 cm^−1^ band is absent in the “after 120 min” spectrum as a result of increased temperature and deacetylation of these groups [60]. The absence of the abovementioned band reveals another band at 1230 cm^−1^, which is assigned to stretching vibrations of bonds (C–C plus C–O plus C=O) from the moiety of more condensed guaiacyl units [61]. Another two bands, with peaks at 1185 cm^−1^ and 1135 cm^−1^, are from the C–O stretching vibration of alcohol groups [62]. Both of them have small shifts for the “after 120 min” spectrum (to 1180 cm^−1^ and 1130 cm^−1^, respectively). These shifts are also the result of the liquid-to-solid phase transition [55]. Also, a significant increase of absorbance is observed for the region of multiple small bands around 1135 cm^−1^ for the last liquid phase spectrum “after 90 min”. This indicates a higher concentration of C–O groups from opened epoxy rings. The broad band with two peaks around 1040 cm^−1^ and 1010 cm^−1^ is assigned to the C–O–C stretching of aryl–O–CH_2_ ethers [63]. The second peak (1010 cm^−1^) has an epoxy ring origin due to a less significant liquid-to-solid decrease of intensity in comparison with its 1040 cm^−1^ counterpart. The 915 cm^−1^ band, which becomes flatter for the “after 120 min” spectrum (compared to other bands with similar original intensity in the liquid phase), is assigned to epoxy ring stretching vibration [64]. The broadband (880–720 cm^−1^) with the highest peak at 830 cm^−1^ (shift to 825 cm^−1^ for solid-state spectrum) indicates the presence of out-of-plane bending vibrations of =C–O from substituted aromatic compounds.

### 2.6. Three-Point Flexural Test and Charpy Impact Test

Flexural modulus (E_f_), maximum flexural strength (σ_fM_), and deformation for maximum flexural stress (ε_fM_) of REF and EXPINE were compared in Table 3. REF exhibits a higher value of flexural modulus and has higher flexural strength. Also, the deformation value is higher for REF, meaning that it is more flexible.

**Figure 8:**
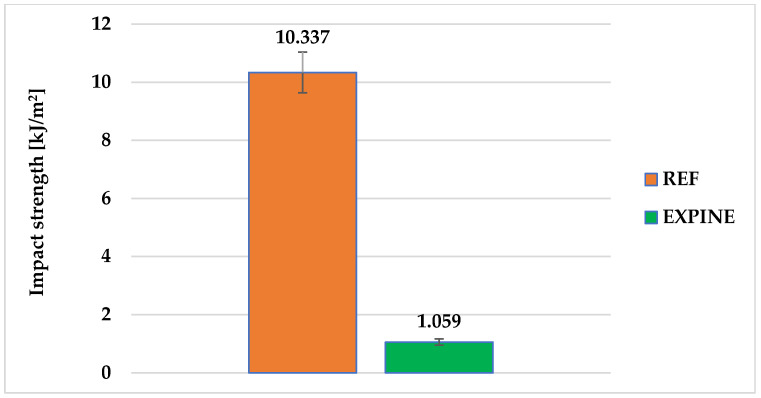
Charpy impact strength results.

Averaged impact strength results of REF and EXPINE compositions are presented in Figure 8. The impact strength value of REF is comparable with other neat epoxy systems [65,66] and is almost 10 times greater than the EXPINE value.

### 2.7. Shore D Hardness

**Figure 9:**
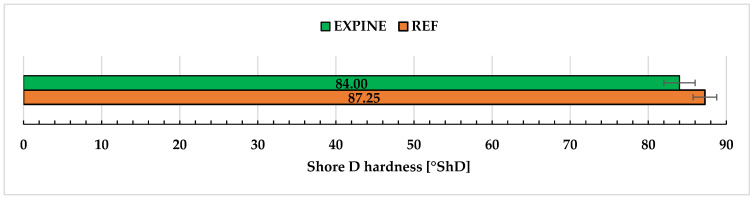
Shore D hardness results.

The Shore D hardness of REF and EXPINE samples were compared in Figure 9. Both compositions show good resistance to deformation by indentation (over 80°ShD), typical for the range of commercial epoxy resins (80–90°ShD). The slightly better performance of REF is not significant as they are within the margin of error, meaning EXPINE could be more competitive in coating applications.

### 2.8. Density Measurements

Density results of REF and EXPINE according to REGLINP function with coefficients of determination (R^2^) were compared in Table 4. REF shows a slightly higher average density, but the difference is within error bars. Both density values correspond to Epidian 5 and TFF values. Also, the coefficients of determination confirm good fitting.

## 3. Discussion

Obtained TPC values of extracts seemingly do not correspond well with TSC values and UV-Vis analysis. While C120 has the highest content of hydroxyl groups attached to aromatic moieties, more polyphenols are found in U30 and U30+C90 extract (same for TSC and UV-Vis analysis). It may result from better extraction of polyphenols rich in hydroxyl groups (like taxifolin) for C120, while UAE extracts provide compounds with fewer substituents.

**Figure 10:**
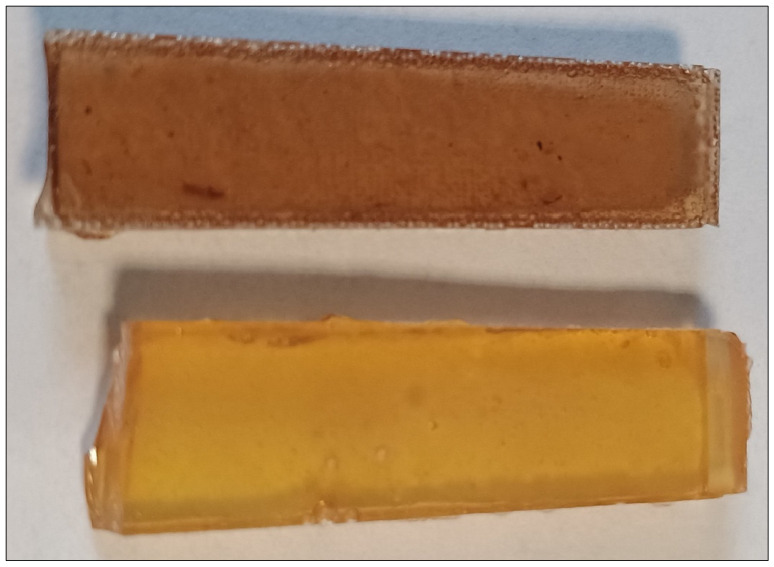
Samples pieces—REF (at the bottom) and EXPINE (at the top).

DSC and ATR FTIR analyses provide two potential causes of EXPINE mechanical limitations—methanol evaporation during gel time (although the sample preparation process was different from the DSC regime) and restricted post-curing reactions (steric hinderance of aromatic groups). Increased porosity of EXPINE as a result of remaining methanol in hardened resin was investigated by density measurement. A slightly higher density of REF corresponds with sample investigation—EXPINE has increased pore content only in the edges area (Figure 10). According to this, porosity is not the only explanation for worse mechanical properties.

More influential is probably limited crosslink density and increased stiffness of the matrix, resulting from the aforementioned steric hinderance of aromatic groups. The relatively high aromatic content of BADGE and phenol derivatives (from TFF) was increased by polyphenolic phytochemicals. In the aspect of mechanical properties composition still needs improvement to be an alternative for relatively competitive BADGE-TFF systems [46]. The addition of more aliphatic segments to the polymer chain is necessary. It can be obtained by different epoxy substrates or amine hardeners with lower aromatic content.

Still, preliminary research was conducted, and results are promising in the context of further work and compared to similar epoxy systems. Flexural modulus (1.86 GPa) and strength (49.5 MPa) are slightly worse than the best ELO-TA system proposed by Korey (over 2 GPa and over 65 MPa, respectively) [29]. Hardness with high Shore D values (84°ShD) is much higher than Shore A values (less than 40°ShA, corresponding to less than 10°ShD) obtained for epoxy with extract fractions as corrosion inhibitors [32].

## 4. Materials and Methods

### 4.1. Preparation of Scots Pine Bark

Bark of Scots pine (*Pinus sylvestris*) was collected in March 2023 around Stryków (51°54′04″ N 19°36′39″ E) in Poland. Selected tree was fallen down due to weather conditions. Bark flakes from the trunk were carefully detached from the trunk and stored in refrigerator. Flakes were grounded before extraction using Bosch TSM6A013B coffee grinder (Bosch, Weissach, Germany). Only a fraction with diameter under 1 mm was used for extraction. The ground bark was stored in refrigerator if not used immediately for extraction.

### 4.2. Extraction of Grounded Bark

For each extraction, 1 g of finely grounded bark (d < 1 mm) was added to flask with 10 mL of methanol (HPLC grade, Honeywell, Charlotte, NC, USA). Methanol was chosen as a compromise between good extraction efficiency, low boiling point, and good compatibility with epoxy systems (unlike water). Two extraction methods—conventional extraction (CE)—mixing with magnetic stirrer at elevated temperature and ultrasound-assisted extraction (UAE) were performed. Heidolph MR-Hei Standard hot plate with magnetic hot stirrer (Heidolph Instruments, Schwabach, Germany) was used for CE (750 rpm for all extracts). UAE was performed in ultrasonic bath Bandelin Sonorex DL 510 H (Bandelin Electronics, Berlin, Germany). Detailed extraction conditions of CE, UAE, and mixed extraction are presented in Table 5.

All obtained extracts were centrifuged, and bark was removed. Extracts without sediment were stored in refrigerator.

### 4.3. Total Phenolic Content Analysis

The total phenolic content (TPC) of pine bark extracts was measured via colorimetric analysis (Folin–Ciocalteu method) [67]. Extracts were diluted with distilled water (1:19, v:v) to obtain absorbance less than 1 (for quantification according to calibration curve). 100 µL of diluted extract, 200 µL of Folin–Ciocalteu reagent, and 3000 µL of sodium carbonate (6.67% solution) were mixed and stored in a dark place for 1 h. Blank sample (100 µL of methanol diluted in distilled water, 1:19, v:v) was prepared in the same way. The absorbance was measured at the wavelength of 765 nm (blank sample as reference) using Specord 50 UV-Vis spectrophotometer (Analytik Jena AG, Jena, Germany). The absorbance of each extract was the mean value of six samples. TPC values were calculated according to calibration curve and expressed as micrograms of gallic acid equivalent per millilitre of extract (µg GAE/mL). Also, the theoretical concentrations of phenol groups (C_PhOH_) were calculated (according to low error level for GAE-based measurements) [68] using equation:

(1) $$C_{PhOH}=(TPC\cdot 3)/M_{GA},$$

where M_GA_ is a molar mass of gallic acid (170.12 g/mol, expressed as µg/µmol).

### 4.4. Total Solid Content

Four extracts (C30, C120, U30, U30+C90) were selected as representatives for determination of solid content. Six samples of each extract were dosed (volume 0.5 mL) with a mechanical micropipette (HTL Labmate Pro LMP1000, Corning HTL SA, Warszawa, Poland) into separate glass weighing bottles with glass caps. Before dosing, bottles with caps were weighted using Sartorius RC210D balance (Sartorius, Göttingen, Germany) with accuracy of 2 × 10^−5^ g. Bottles were closed with cap immediately after dosing to minimise evaporation of methanol (also for verification of dosing repeatability) and weighed again. Samples were left for 24 h under fume hood for air drying (without the heat) and then weighted again (all methanol evaporated). Results were calculated according to the equation:

(2) $$TSC=\frac{m_{BE}-m_{B}}{m_{BS}-M_{B}}\cdot 100\%,$$

where: m_BE_—mass of bottle with extract, m_B_—mass of empty bottle, and m_BS_—mass of bottle with solids after evaporation (all masses take into account mass of glass cap). All TSC values were expressed as a percentage.

### 4.5. UV-Vis Identification of Phytochemicals

Eight extracts (C30, C60, C90, C120, C150, C180, U30, and U30+C90) were selected for UV-Vis analysis. Selected extracts were diluted in methanol (HPLC grade, Honeywell, Charlotte, NC, USA) to 0.33% concentration, placed in quartz cuvette (thickness—1 cm), and analysed using Specord 50 single-beam spectrophotometer (Analytik Jena AG, Jena, Germany). A blank sample containing only methanol was used as a reference and spectra of all extracts were processed with blank subtraction using WinAspect 2.3.1.0. software (Analytik Jena AG, Jena, Germany). Absorbance values were compared for the wavelength range 205–355 nm (no peaks observed for higher wavelengths).

### 4.6. DSC Analysis of Curing Process

The DSC measurements were carried out using Linseis Chip-DSC 100 calorimeter (Linseis Messgeraete GmbH, Selb, Germany). Each composition was mixed and placed in 100 µL aluminium crucible (c.a 1/3 of crucible’s volume) with a lid. The samples were heated twice (1st and 2nd cycle) from 35 °C to 300 °C with heating speed 10 °C/min. REF was prepared according to producer’s suggestion—BADGE resin Epidian 5 (Sarzyna Chemical, Nowa Sarzyna, Poland) was mixed with Mannich base (MB) hardener TFF (Sarzyna Chemical, Nowa Sarzyna, Poland), containing triethylenetetramine (TETA), phenol and oligomers of the above with formaldehyde. Resin to TFF hardener ratio for REF was 100:26 (*w*/*w*). EXPINE composition was modified by replacing half the weight amount of TFF hardener with selected methanolic extract with the highest TPC (C120). Resin to TFF hardener to C120 extract ratio for EXPINE was 100:13:13 (*w*/*w*/*w*). More detailed information about BADGE resin and MB hardener is presented in Table 6.

### 4.7. ATR FTIR Analysis of Curing Process

Attenuated total reflection (ATR) Fourier transform infrared spectroscopy (FTIR) was the method selected for investigation of curing process. The analysis was performed using a Nicolet iS5 FT-IR spectrometer (Thermo Fisher Scientific, Waltham, MA, USA) with iD7 ATR Accessory (with ZnSe crystal). EXPINE composition (resin to TFF to selected pine bark extract ratio: 100:13:13, *w*/*w*/*w*) was analysed during curing process from room temperature to 100 °C. Samples for analysis were taken every 30 min and immediately measured. A total of 16 scans were taken for each spectrum, with a resolution of 4 cm^−1^. All spectra were processed using Advanced ATR Correction and Automatic Baseline Correction tools in the OMNIC 9.11.727 software (Thermo Fisher Scientific, Waltham, MA, USA).

### 4.8. Preparation of Samples for Mechanical Tests

The rectangular (80 mm × 10 mm × 4 mm, without notch) samples of both compositions (REF and EXPINE) were prepared for mechanical tests (Charpy impact test and three-point flexural test). EXPINE composition was prepared in two steps. Pine bark extract was mixed with epoxy in the first step, and then commercial hardener was added. Both compositions, after mixing were poured into silicone moulds and left for 24 h at room temperature (curing) and then for 5 h at 100 °C (post-curing). The samples were demoulded carefully and prepared for mechanical tests (removal of overflowed edges). All samples were stored at a temperature of 20 °C and air humidity of 45% before mechanical tests.

### 4.9. Three-Point Flexural Test

Zwick/Roell 1435 universal testing machine (Zwick/Roell, Ulm, Germany) with a three-point flexural test configuration (two supports and loading part) was used at a testing speed of 2 mm/min. Six rectangular samples from Section 2.7. of each composition were tested, and the results were averaged.

### 4.10. Charpy Impact Test

Cometech QC-639P/Q universal impact tester (Cometech, Taichung, Taiwan) with 2J pendulum hammer and Charpy vise was used at a striking speed of 2.9 m/s. Six rectangular samples from Section 2.7. of each composition were tested, and results were averaged.

### 4.11. Shore D Hardness

The hardness of twenty points on the surface of the samples was measured manually for each composition. Sauter HBD 100-0 Shore D hardness tester (Sauter, Freiburg, Germany) with an accuracy of 1°ShD was used. The accuracy of the device was checked using a standard every five measurements. Results were averaged.

### 4.12. Density Measurement

Small pieces of samples from mechanical tests were weighted using Sartorius RC210D balance (Sartorius, Göttingen, Germany) with an accuracy of 2 × 10^−5^ g. Then, volume changes after immersion of samples in water were read from the measuring cylinder scale (accuracy of 0.5 mL). Obtained values were used for density calculations (d = m/V). Density values and standard deviations were calculated for both compositions (each from 12 samples) using REGLINP function in Microsoft Excel (Microsoft, Redmond, WA, USA).

## 5. Conclusions

Scots pine bark methanolic extract revealed the expected potential as a “green” alternative to commercial hardeners with high TPC value and the presence of other functional groups. Evaluation of different extracts (TPC, TSC, UV-Vis analysis) helped to choose the most representative extract for further tests. EXPINE composition with selected C120 extract was investigated by ATR-FTIR and DSC analysis, and the replacement of half the amount of TFF hardener was effective in terms of the curing process. EXPINE, as the solid sample was also evaluated by mechanical tests, being less competitive in terms of flexural and impact strengths but more comparable to coating (high Shore D hardness).

## Figures and Tables

**Table 1:** Total phenolic content of Scots pine bark extracts and calculated concentration of phenolic groups (the results are expressed as mean ± standard deviation).

No.	Extract Name	TPC [µg GAE/mL]	C_PhOH_ [µmol/mL]
1	C30	2849.9 ± 67.3	50.3 ± 1.2
2	C60	2890.1 ± 72.1	51.0 ± 1.3
3	C90	2598.7 ± 59.0	45.8 ± 1.0
4	C120	3670.2 ± 68.4	64.7 ± 1.2
5	C150	2804.1 ± 80.3	49.4 ± 1.4
6	C180	2784.4 ± 64.9	49.1 ± 1.1
7	U15	1981.1 ± 58.5	34.9 ± 1.1
8	U30	2738.9 ± 60.7	48.3 ± 1.0
9	U45	2264.7 ± 71.1	39.9 ± 1.1
10	U60	2484.2 ± 82.5	43.8 ± 1.3
11	U15+C105	2461.6 ± 76.4	43.4 ± 1.3
12	U30+C90	2813.7 ± 59.7	49.6 ± 1.0

**Table 2:** Total solid content of selected Scots pine bark extracts (the results are expressed as mean ± standard deviation).

Extract Name	C30	C120	U30	U30+C90
**TSC [%]**	1.6714 ± 0.0155	1.8534 ± 0.0455	1.8693 ± 0.0311	2.0144 ± 0.0411

**Table 3:** Results of the three-point flexural test.

	E_f_ [MPa]	σ_fM_ [MPa]	ε_fM_ [%]
REF	2690 ± 80	91.7 ± 5.5	3.41 ± 0.24
EXPINE	1862 ± 59	49.5 ± 3.4	2.66 ± 0.19

**Table 4:** Density values according to REGLINP function.

	Density [g/mL]	R^2^
REF	1.177 ± 0.014	0.9990
EXPINE	1.163 ± 0.020	0.9982

**Table 5:** Detailed extraction conditions.

No.	Extract Name	Extraction Time [min]			Temperature of Extraction [°C]	
		CE	UAE	Total	CE	UAE
1	C30	30	-	30	60	-
2	C60	60	-	60	60	-
3	C90	90	-	90	60	-
4	C120	120	-	120	60	-
5	C150	150	-	150	60	-
6	C180	180	-	180	60	-
7	U15	-	15	15	-	25
8	U30	-	30	30	-	25–30 *
9	U45	-	45	45	-	25–35 *
10	U60	-	60	60	-	25–40 *
11	U15+C105	105	15	120	60	25
12	U30+C90	90	30	120	60	25–30 *

* Temperature during UAE process increased slightly for longer extraction times (about 5 °C for each additional 15 min).

**Table 6:** Detailed information about selected epoxy resin and amine hardener.

	Viscosity (25 °C) [mPa ∙ s]	Density (20 °C) [g/cm^3^]	Epoxy Value (E)/ /Amine Value (A)
Epidian 5	20,000–30,000	1.18–1.19	E: 0.48–0.51 mol/100 g
TFF	max. 10,000	1.15–1.20	A: 500–700 mg KOH/g

## Data Availability

The data presented in this study are mostly openly available. DOI addresses are provided in the References Section.
